# Biomimetic post-capillary venule expansions for leukocyte adhesion studies

**DOI:** 10.1038/s41598-018-27566-z

**Published:** 2018-06-19

**Authors:** Bryan L. Benson, Lucy Li, Jay T. Myers, R. Dixon Dorand, Umut A. Gurkan, Alex Y. Huang, Richard M. Ransohoff

**Affiliations:** 1 Department of Pathology, Case Western Reserve University School of Medicine, Cleveland, OH 44106 USA; 2 Case Western Reserve University School of Medicine, Cleveland, OH 44106 USA; 3 Department of Pediatrics, Case Western Reserve University School of Medicine, Cleveland, OH 44106 USA; 4 Mechanical and Aerospace Engineering Department, Case Western Reserve University, Cleveland, OH 44106 USA; 5 Department of Biomedical Engineering, Case Western Reserve University School of Engineering, Cleveland, OH 44106 USA; 6 Angie Fowler AYA Cancer Institute, University Hospitals Rainbow Babies & Children’s Hospital, Cleveland, OH 44106 USA; 7 Biogen, Cambridge, MA 02142 USA; 8 Present Address: Third Rock Ventures, Boston, MA 02116 USA

## Abstract

Leukocyte adhesion and extravasation are maximal near the transition from capillary to post-capillary venule, and are strongly influenced by a confluence of scale-dependent physical effects. Mimicking the scale of physiological vessels using *in vitro* microfluidic systems allows the capture of these effects on leukocyte adhesion assays, but imposes practical limits on reproducibility and reliable quantification. Here we present a microfluidic platform that provides multiple (54–512) technical replicates within a 15-minute sample collection time, coupled with an automated computer vision analysis pipeline that captures leukocyte adhesion probabilities as a function of shear and extensional stresses. We report that in post-capillary channels of physiological scale, efficient leukocyte adhesion requires erythrocytes forcing leukocytes against the wall, a phenomenon that is promoted by the transitional flow in post-capillary venule expansions and dependent on the adhesion molecule ICAM-1.

## Introduction

The leukocyte adhesion cascade, by which peripheral blood immune cells exit from flowing blood and migrate into tissues, is highly constrained by biological regulation, statistics, and biophysics.

First, molecular regulations on the cellular level constrains the efficiency of leukocyte adhesion. Defects in integrin expression^1–3^, selectin ligand sialylation^4^, or integrin affinity upregulation^5,6^ all lead to severe functional immunodeficiencies, while excessive leukocyte adhesion and accumulation is a contributor to numerous disease states, classically autoimmunity, but also including atherosclerosis^7^, acute lung injury^8^, and sickle cell disease^9^. This regulation occurs with high statistical efficiency. In healthy physiology, 25 billion leukocytes traverse the 100,000 kilometers of vasculature in the body, one circuit per minute, with significant adhesion occurring only in homeostatic sites such as bone marrow, high endothelial venules of secondary lymphoid sites, thymus, spleen, and liver^10–14^.

The biophysical context in which these processes are regulated is also challenging. More than half of the vasculature length is made up of capillaries - vessels that are physically smaller than the 8–15 micrometer diameter of leukocytes - where adhesion would result in plugging of the capillary blood flow and local ischemia. Yet, given the speed of blood flow, adhesive interactions between leukocytes and endothelium must occur within fractions of a second^15^ for any adhesion to be spatially relevant. Despite these challenges, leukocyte adhesion is largely restricted to post-capillary venules (PCVs)^16,17^, vessels of about 20–50 micrometers in diameter that immediately follow capillaries.

Such efficient regulation on a single-cell basis in a stochastic biological system requires multiple overlapping factors to achieve. For example, while shear stress has a significant influence on leukocyte adhesion, it alone is not sufficient to predict the locations where such adhesion occurs^18^. Likewise, while endothelial cell adhesion molecule expression is maximal in PCVs of 25 micrometer diameter^19^, it is not constrained to zero in capillaries. Consequently, multiple levels of regulation may act in a coordinated fashion to control leukocyte adhesion. *In vivo*, inflammation triggers multiple coordinated changes, such as increasing adhesion molecule^20^, selectin^21^, and arrest chemokine expression^22^, the endothelial barrier becoming more permeable to inflammatory signals from the parenchyma^23^, vascular geometry changing from more capillary-like to more PCV-like^24,25^, and erythrocyte aggregation promoting margination of leukocytes to the vascular wall^26,27^.

Therefore, to maximize physiological relevance, for the study of leukocyte adhesion, microfluidic models should closely mimic the scale and fluid dynamics of *in vivo* vessels. Currently, the majority of microfluidic leukocyte adhesion assays use device channels with a height of 100 micrometers or larger^28–30^. While microfluidic designs featuring *in vivo* capillary and PCV scale have been used successfully to investigate scale-dependent effects on cell migration, mechanics, and capillary transit^31–34^, designs specifically aimed at investigating the process of leukocyte capture from flowing blood are much fewer in number^30,35^. The common feature of these smaller channel devices is low numbers of observed adherent cells, precluding their use to quantify the effect of perturbations on leukocyte adhesion. Such low numbers are counter to the predictions of some physical models that postulate higher leukocyte adhesion probability in smaller channels^36^. However, other models show that the smaller vessels with decreasing channel height resulted in significant partial occlusion by leukocytes at vessel sizes below 50 micrometers, increasing peak shear stress on the cell surface^37^, and shrinking cell-surface contact area^38^. Finally, the small perimeter of these near-physiological channels limits the surface area available for leukocyte interaction with the channel surface.

To address these challenges, we took a two-pronged approach: (1) directly visualize leukocyte arrest behavior *in vivo* to design microfluidic devices that recapitulate the *in vivo* forces on leukocytes, and (2) leverage simple computer vision techniques and repeating device designs to overcome the statistical challenges of low numbers and variable leukocyte adhesion events in microchannels.

*In vivo* imaging of leukocytes suggested that arrest was most prominent at areas of sudden vessel volume expansion. We hypothesized that mimicking such expansions would increase numbers of adherent cells in our microfluidic assay. We expected that these leukocytes would arrest at the channel side walls due to margination induced by such expansions^39^. Early experiments suggested that extensional stress at expansions was critical. Therefore, we used computational fluid dynamics (CFD) simulations to create devices that approximate the rheological stresses experienced by leukocytes in PVC expansions.

Here, we introduce devices and an analysis pipeline that makes possible statistically valid studies of leukocyte adhesion in microfluidic channels of physiological scale. We use these devices to investigate the effects of rheological stresses at the capillary to PCV transition on leukocyte adhesion probability, with the hypothesis that extensional stresses will increase leukocyte adhesion probability. Our results indicate that interactions with other flowing cells within the microchannel, which are governed by rheological stresses, rather than rheological stresses *per se*, most strongly determine leukocyte adhesion probability.

## Results and Discussion

### Observation and simulation of *in vivo* post-capillary venules

**Figure 1:**
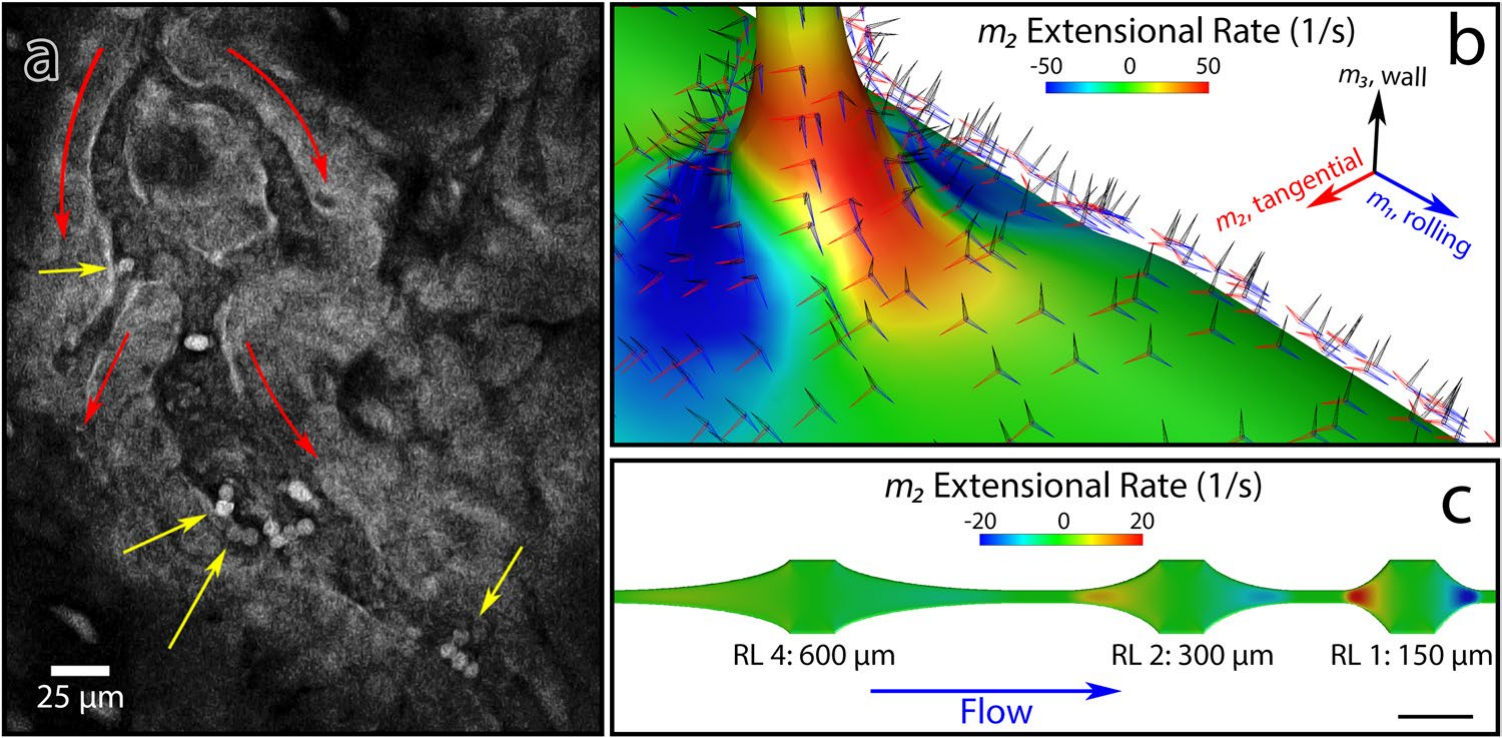
Mimicry of *in vivo* extensional stress (**a**) A snapshot of *in vivo* two-photon microscopy imaging of inflamed pial vessels in a ubiquitin-GFP mouse (Supplemental Movie 1). :Flow direction. :Stably adherent leukocytes appear to be preferentially located after volume expansions, either at capillary to post-capillary venule (PCV) transition (top left arrow) or at expansions within a vessel (other three arrows). Scale bar: 25 micrometers. (**b**) Computational fluid dynamics simulation of capillary to PCV transition, demonstrating wall-and-rolling relative tensor basis, and extensional stress incurred as the leukocyte enters the PCV. (**c**) CFD of biomimetic device that varies extensional stress by varying expansion length, while keeping shear stress equivalent. Scale bar in lower right shows 250 micrometers. Numbers indicate hyperbola lengths, with relative length (RL) = hyperbola length/150 micrometers. A shorter length corresponds to a higher extensional rate.

To directly observe the patterns of leukocyte adhesion *in vivo*, we implanted cranial windows into Ubiquitin-GFP mice^40^, injected vessel dye, and used two-photon laser scanning microscopy (2PLSM) to capture the behavior of leukocytes and endothelial cells in real time. We first sought to make a systematic study, collecting 455 × 455 × 55 micrometer fields of view every 20 seconds (3D + time). However, while we were successful in capturing overall leukocyte adhesion under different conditions, leukocyte crawling motility was too fast relative to the sizes of vessels for precise adhesion locations to be identified as a function of rheological stresses. We therefore switched to real-time observation of leukocyte-endothelial interactions using resonance scanning and a 2D + time scanning mode. With this method, it became apparent that leukocytes were preferentially adhering in regions of expansion (Fig. 1a, Supplemental Movie 1).

To mimic these expansions *in vitro*, we turned to CFD simulations of a stereotypical capillary-PCV junction, as we were not able to directly measure three-dimensional flow characteristics with sufficient spatiotemporal resolution *in vivo*. We chose to model a stereotypical capillary to PCV intersection using a 90-degree intersection between an 8 micrometer capillary and a 25 micrometer PCV. In this model, we used as inputs established volume rate of flow vs. diameter data from human conjunctival vessels^41^, assuming conservation of mass and adjusting the diameter of the pre-junction PCV to compensate for the capillary side flow. Resultant shear stress and pressures were close matches for reported physiological values. We rotated the simulation outputs to a leukocyte-centric coordinate system (Supplemental Equations 1) at each location: *m*_*1*_, the direction along which a leukocyte would roll, *m*_2_, the direction along the wall, and *m*_3_, the axis of contact between the leukocyte and the wall. For extensional strain, which we took as a quantification of the expansion effect, the rate in the *m*_2_ direction best captured the expansion phenomenon as observed *in vivo*, with a defined region of extension occurring at the exit from capillary to PCV. Figure 1b shows these extensional rates experienced by leukocytes, as well as the rotation of basis.

### Design of the biomimetic microfluidic devices

We created three devices to systematically investigate the effect of channel expansions on leukocyte adhesion, progressing from near-physiological to physiological scale (Supplemental Fig. 1).

The first device (Supplemental Fig. 1A) featured a circular manifold with 54 channels, each containing one region of interest (ROI), taking off directly from a circular manifold. Due to issues with clogging of channel inlets under certain conditions, pre- and post- expansion widths were set at a constant 50 and 250 micrometers, respectively. Channel height was set at 25 micrometers. We used hyperbolic expansions terminated at an angle significantly less than 90 degrees in order to avoid the confounding issues of stagnation points or vortices immediately post-expansion. Following each expansion was a long straight section, over which time the effects of the expansion could relax, so that adhesion could be investigated under established flow that represents the *status quo*.

We made two major modifications in the second device (Supplemental Fig. 1B). First, we added ROIs in which the relative strength of the expansion is varied by varying the length of the expansion, keeping all other parameters constant (Fig. 1c). These ROIs were added in series, three to a channel in counterbalanced order, for a total of 240 ROIs per device. The shortest (relative length 1, at 150 micrometers) expansion provided 20 1/s extensional strain rate in whole blood, whereas the longest (relative length 4, at 600 micrometers) expansion provided 5 1/s. The intermediate (relative length 2, at 300 micrometers) expansion provided 10 1/s. This allowed us to investigate the effects of expansion independent of shear stress, which is identical at 2.4 dyne/cm^2^ under typical operating pressure across all designs. The second major change was a branching inlet pattern using the planar adaptation of Murray’s law^42^. This modification dramatically reduced the occurrence of channel inlet blockade.

In the third device (Supplemental Fig. 1C) we investigated whether the reduction in clogging from the branching inlet pattern would allow us to use channels of physiological scale. These devices were 20 micrometers in height, featuring 16 micrometer narrow channels to expansions of 48 and 60 micrometers. These expansions were arrayed two per channel, in counterbalanced order, for a total of 512 ROIs per device.

With the emphasis placed on throughput, the assembled chips consisted of a single layer of PDMS bonded to cover glass, coated with immobilized adhesion molecule ICAM1, chemokine CXCL12, and glycosaminoglycan (GAG)-bearing proteoglycan SDC4. These molecules were chosen due to the near-uniform expression of the cognate integrin LFA-1 and chemokine receptor CXCR4 on peripheral blood leukocytes. SDC4 was chosen as the most representative proteoglycan expressed on endothelial cells. Gravity was used to drive flow, using P1000 filter pipet tips as reservoirs set to a specific fluid height.

### Creation of an automated data analysis workflow

**Figure 2:**
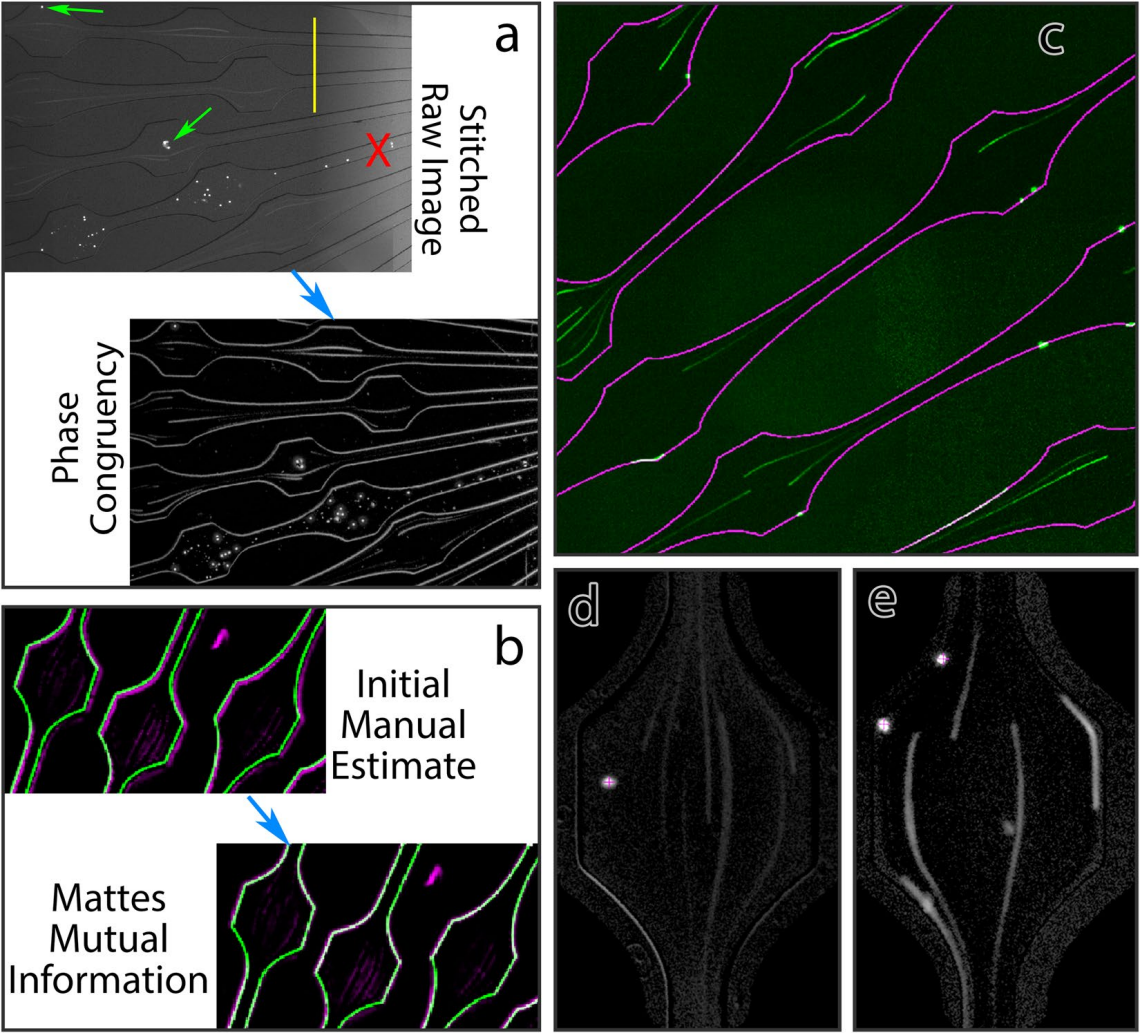
Data handling workflow. (**a**) Raw data, demonstrating goals and challenges. Top left: tiled microscopy images show adherent leukocytes , but also have large inhomogeneities due to differences in background illumination between tiles . Additionally, some lanes are blocked and must be excluded from further analysis . Bottom right: the same data after phase congruency calculation. This isolates the important features, while removing effects of background inhomogeneity. (**b**) Registration of cleaned phase contrast images to the template. Top left: initial manual rigid registration results in mismatches between the phase congruency map (magenta) and the device template (green). Affine registration using Mattes mutual information results in near-perfect alignment between the data and the template (bottom right). (**c**) Raw data from cells run in the presence of RBCs (green) superimposed with the device template (magenta), demonstrating the quality of the resultant alignment. Wall-adherent and rolling leukocytes colocalize with the wall delineated on the template. (**d**) Example of ROI from media (LCIS) condition. (**e**) Example of ROI from RBC condition, demonstrating sidewall-adherent leukocytes, a center adherent leukocyte, flowing leukocytes, and rolling leukocytes.

After sample and device preparation time, each experimental run required only 15 minutes to complete. Therefore, data acquisition throughput met its practical limit only by the constraints of device construction steps such as cleaning and bonding. To generate statistically meaningful data from this volume of raw imaging data required workflow automation. Of particular interest to us was the ability to spatially register each leukocyte so that the patterns of cell-specific leukocyte adhesion probabilities in response to rheological stresses could be observed. A tile-scanning microscope was used to image the entire chip surface at the beginning and end of collection. Due to out-of-plane fluorescence from cells in the inlet region and blockade of fluorescence in experiments with red blood cells, there were significant inhomogeneities in background fluorescence between tiles (Fig. 2a). Nevertheless, channel edges were still clearly discernible. Attempts to use the raw fluorescence data to register devices to the template failed. To allow registration, we consulted the computer vision literature and Kovesi’s method of calculating phase congruency^43^, which, unlike other edge detection methods, is spatially faithful and contrast-invariant. After initial filtering, phase congruency images efficiently detected channel edges as well as flowing and adherent leukocytes (Fig. 2a). This image could further be subdivided into leukocytes versus channel edges by multiplying the phase congruency estimate by a constant and the sine of the phase angle: leukocytes, as bright objects, have phase angles approaching π/2, whereas the channel edges are dark, with phase angles approaching -π/2.

With divided phase congruency images in hand, we could then efficiently align the microscopy data to our CAD device designs. This started with a rigid manual alignment, which was easily performed conjointly with visual inspection of the data to ensure that there were not major abnormalities such as chip delamination or large manufacturing defects. During this time, lanes blocked with PDMS debris were marked for exclusion from further analysis (Fig. 2b, refer also to Fig. 1a). Overall, 240 out 21,030 ROIs (1.14%) were excluded in this manner. Of these, exclusion rate was highest for the first device, not designed with Murray’s law in mind, with 94/2646 ROIs excluded (3.55%).

Manual alignment provided an initial estimate but resulted in minor registration errors. Due to the deformability of PDMS, devices frequently exhibited slight stretch from one side to the other. This stretch is not significant enough at the level of a single ROI to affect fluid dynamics but is significant enough across a millimeters-wide device to affect registration. Therefore, we turned to affine registration using Mattes mutual information, which generally resulted in excellent alignment between the microscopy data and the device template (Fig. 2b,c). Once aligned to a common template space, collecting ROI-by-ROI data across devices and participants became trivial, applying a simple program to align ROIs to each other using known geometric transformations from the device layout.

### Automatic cell segmentation

**Figure 3:**
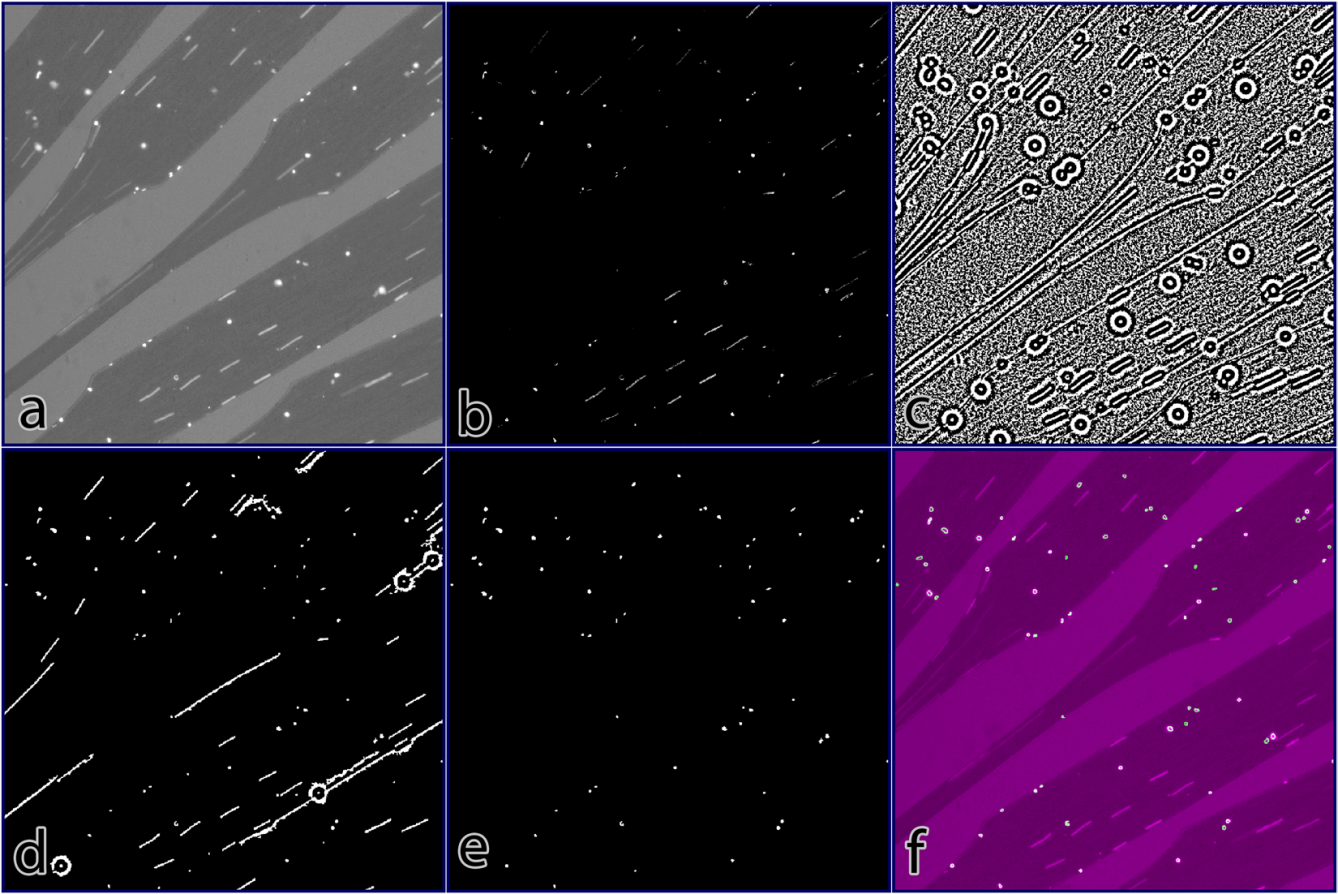
Automated Cell Segmentation. (**a**) Raw data, demonstrating background, ROIs, adherent, and flowing leukocytes. (**b**) Phase congruency of data. Note discontinuities in phase congruency of flowing leukocytes. (**c**) Phase angle of data. This eliminates discontinuities but is too noisy to use as standalone. (**d**) Initial segmentation, using seed points from phase congruency and region growing using phase angle to reconstruct the image. (**e**) Segmentation after morphological filtering to remove flowing and rolling cells, filter artefacts, and platelets. (**f**) Overlay of segmented cells (outlined in green) over the raw data (magenta).

The volume of ROIs to analyze precluded manual counting from being practical. We therefore developed a method for automatic segmentation, outlined in Fig. 3. First, raw images are high-pass filtered to isolate cells, which are high frequency data, from low frequency data such as channel geometries and background illumination. Then phase congruency and phase angles are calculated using two separate wavelengths. Phase congruency at the shorter wavelength identifies cells (Fig. 3b) but does not robustly identify the full extent of flowing cells, preventing morphological analysis from distinguishing flowing vs. adherent cells. To circumvent this issue, we combined this information with the phase angle (Fig. 3c) calculated at a longer wavelength. This robustly identifies the extent of flowing cells but is too sensitive to noise to be used as a standalone segmentation method. The resulting reconstruction (Fig. 3d) using both data robustly identifies cells at a range of intensities and identifies the full contiguous regions of flowing cells. From this starting point, simple morphological filtering then isolates adherent cells (Fig. 3e,f). This algorithm performed better than trained human operators. Overall correspondence was 85% for the algorithm vs. human operators in spot checks, but subsequent re-examination demonstrated that this was attributable to deterioration of human counting performance over several continuous hours.

### Efficient adhesion in expansions requires cell-cell forcing

With the data acquisition and analysis pipeline established, we performed leukocyte adhesion assays using freshly-isolated peripheral blood mononuclear cells (PBMC) from healthy human donors, using immobilized recombinant human CXCL12 (rhCXCL12) and recombinant human Fc-ICAM1 fusion protein (rhFc-ICAM1) as substrates. To maintain physiological pH, cells were resuspended in live cell imaging solution (LCIS), a HEPES buffered, calcium-containing saline, plus bovine serum albumin (BSA) and a nondiabetic amount of D-glucose (50 mM).

**Figure 4:**
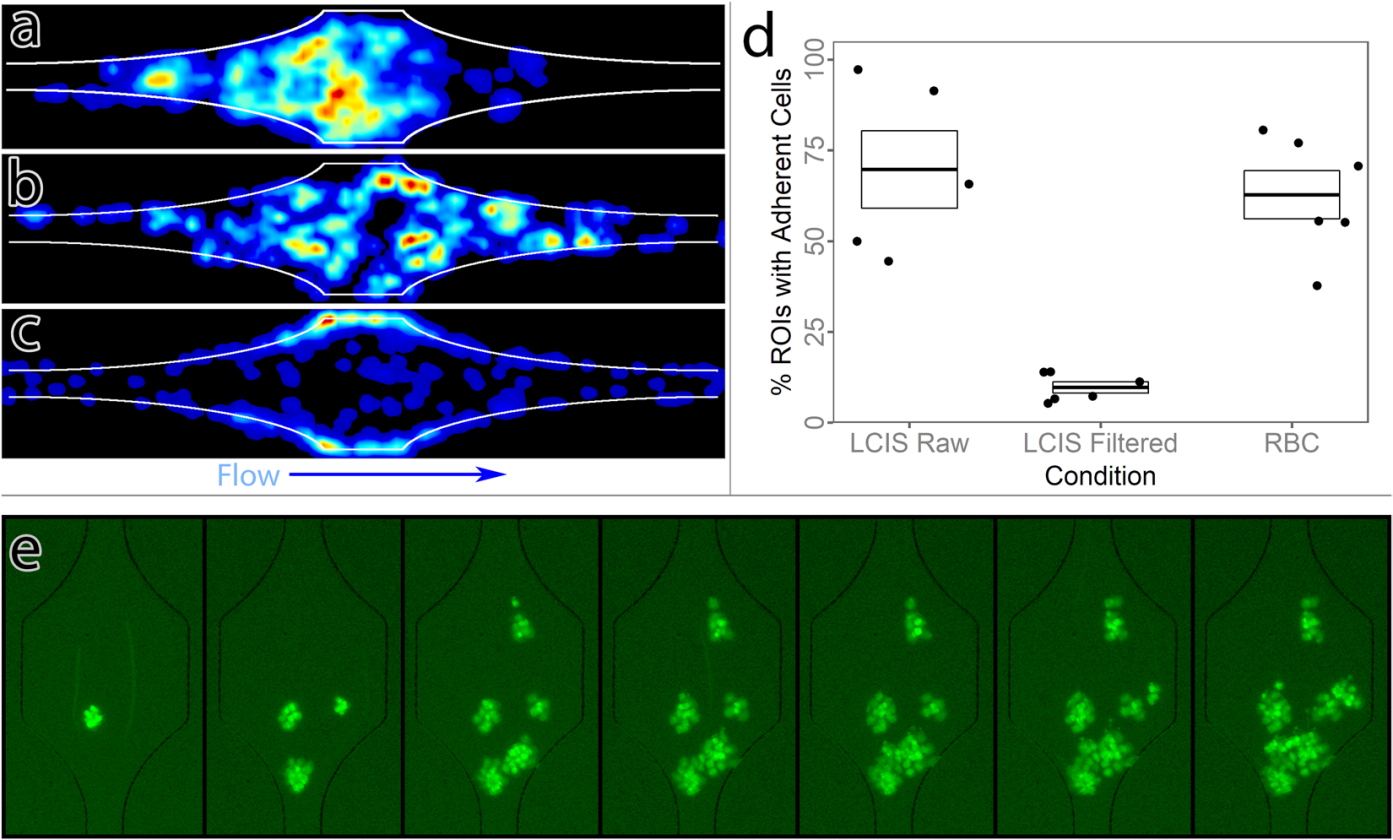
Efficient leukocyte adhesion requires cell-cell forcing at physiological scale. (**a**–**c**) Heatmaps showing leukocyte adhesion probability distribution across length 4 ROIs. (**a**) Unfiltered leukocytes in media, demonstrating a bias towards the front of the ROI. (**b**) Filtered leukocytes in media. (**c**) Filtered leukocytes in media reconstituted with erythrocytes at physiological hematocrit, demonstrating redistribution to the sidewalls. (**d**) Overall adhesion probabilities for leukocytes in the three conditions, demonstrating a profound lack of adhesion for filtered leukocytes in media. Each point represents averaged data from one participant, N = 5 donors. Boxes indicate mean and standard error. (**e**) Leukocyte adhesion over time with unfiltered leukocytes. Time progresses from left to right in 5-minute increments. Initial adhesion is seeded by small clumps or doublets, which form nuclei for later secondary capture of leukocytes.

Initial experiments with typical methods of cell preparation used in the field, with isolated PBMC in otherwise cell-free media showed robust adhesion focused in the initial expansion regions of the ROIs (Fig. 4a,d, left), but close inspection of time lapse imaging data revealed that the majority of adhesion was driven not by single leukocytes, but by doublets or larger aggregates of cells, which formed nucleation points for further secondary capture (Fig. 4e). Under these conditions, adhesion probability did not have a linear response with time, but rather was exponentially increased by adhesion of a prior leukocyte^44,45^, a process termed “secondary capture”.

Although clumping adhesion has long been described for cancer metastasis, and we observed occasional leukocyte doublets in circulation by intravital 2PLSM, in general leukocyte adhesion is not driven by aggregates *in vivo*. Thus, we suspected that the adhesion as observed in our device was most likely an artifact of the cell preparation method. To test this, we passed the prepared cells through a filter to enforce a single cell suspension. Under these conditions, clumping adhesion and secondary capture were all but eliminated. Interestingly, the apparent biasing towards the expansion was eliminated, but so was overall adhesion (Fig. 4b,d, center).

From these data, we hypothesized that in such small channels, cell-cell forcing due to collisions may be necessary to increase contact area and overcome the effects of focally high shear stress, allowing firm adhesion to be established. *In vivo*, leukocytes do not circulate in free plasma but are constantly surrounded in large numbers by red blood cells (RBCs). Collisions with RBCs within the vasculature could create the necessary strong forces on leukocytes to allow adhesion. We therefore performed leukocyte adhesion assays on filtered PBMCs to which RBCs were reintroduced at 50% of the total volume. These RBCs were isolated by centrifugation, resulting in a packed volume approximately 10% higher than true clinical hematocrit: thus we expect the 50% volume ratio to represent a hematocrit of approximately 45%, which is within physiological range, and corresponds to the value we used for our CFD simulations. Re-introduction of RBCs restored leukocyte adhesion (Fig. 4d) while strongly shifting probability density from the center of the channel to the side walls (Fig. 4c). On an ROI-by-ROI basis, this also all but eliminated secondary capture effects and thus decreased variability in mean adhesion between ROIs.

The inclusion of RBCs also led to the induction of ICAM-1 mediated slow rolling, which is visible on the sidewalls of the device (Supplemental Movie 2), an effect consistent with predictions of computational studies^46,47^.

### Expansion-induced leukocyte adhesion is dependent on sidewall contact and ICAM-1

To further characterize the effect of the expansion on leukocyte adhesion, we investigated leukocyte adhesion patterns in ROIs in which we coupled an expansion to a long, straight section over which the expansion effect would be extinguished. In this platform, we observed leukocyte adhesion in response to a factorial combination of adhesion molecule ICAM1, chemokine CXCL12, and heparan sulfate proteoglycan SDC4, which is widely expressed by endothelial cells and can bind and immobilize CXCL12^48,49^. In simple coating experiments using AlexaFluor 647 labeled CXCL12^50^, we observed that SDC4 increased the amount of immobilized CXCL12, and changed the distribution from uniform to somewhat punctate.

Leukocyte adhesion probability in all the ICAM-1 containing conditions are demonstrated in 5A. As in the shorter channel, sidewall adhesion was dominant. However, two broad trends were noticeable. First, adhesion was most robust on the sidewalls immediately following the expansion. Second, adhesion in the center of the channel remained steady, or even slightly increasing over the length of the ROIs.

**Figure 5:**
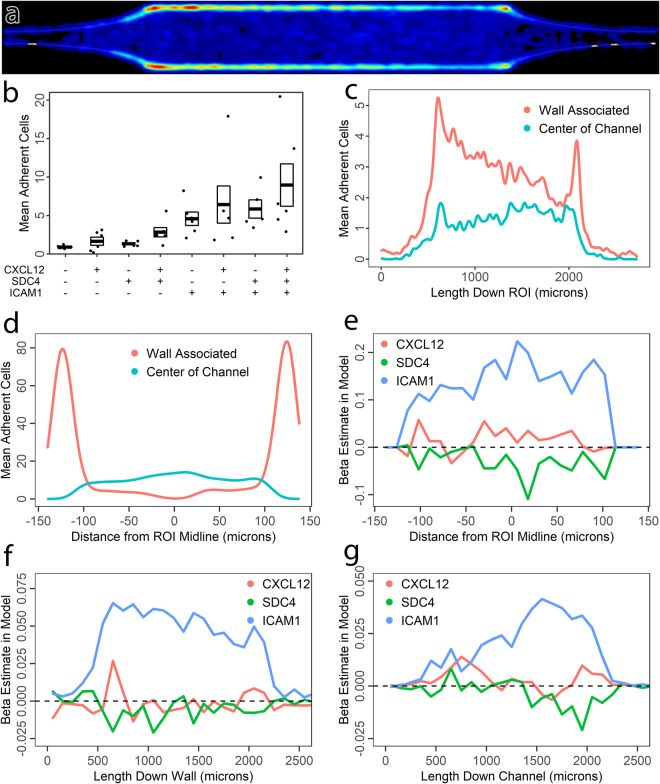
Spatial statistics of leukocyte adhesion as a function of coatings. (**a**) Leukocyte adhesion distribution heatmap for all ICAM-1 containing conditions in the straight channel device (Supplemental Fig. 1A). Flow direction is left to right. These consist of a length 4 expansion and an extended straight section to capture relaxation of the expansion effect. Wall associated adhesion peaks immediately after the initial expansion, and again at the collision-inducing point where the expansions end. Channel center adhesion subtly increases over the length of the channel. (**b**) Mean number of adherent cells per ROI in response to factorial combinations of CXCL12, SDC4, and ICAM1 in the coating, demonstrating additive effects. Each point represents mean numbers for a single participant, N = 6 donors. Boxes indicate mean and standard error. (**c**) Sub-ROI quantification using a mask for wall-associated vs. center of channel leukocytes as a function of flow distance along the ROI, demonstrating relaxation of the expansion effect for wall-associated but not channel center leukocytes. Wall associated adhesion probability peaks immediately after the expansion, then declines across the ROI until the junction with the constriction induces further collisions between erythrocytes and leukocytes, generating a second peak of adhesion. In contrast, channel center adhesion gradually increases across the length of the channel. (**d**) Validation of the sub-ROI quantification method by quantifying across the cross section of the ROI: Wall associated adhesion is restricted to the sidewalls, whereas channel center adhesion peaks in the channel center. (**e**) Linear model fits across the ROI cross section for the relative contributions of CXCL12, SDC4, and ICAM1 to adhesion. As expected, the contribution of adhesion molecule ICAM1 is most significant in the channel center, where shear stress is highest. (**f**) Linear model fits along the ROI for coating contributions to leukocyte adhesion on the sidewall. The initial spike of adhesion at the expansion is strongly ICAM-1 dependent. (**g**) Linear model fits along the ROI for leukocyte adhesion in the center of the channel. Adhesion immediately after the expansion is dependent on all three coatings, whereas ICAM-1 becomes more dominant as the expansion effect relaxes.

As Fig. 5b demonstrates, overall adhesion was dependent on ICAM1, *F*(1,38) = 60.9, *p* < 0.001, SDC4, *F*(1,38) = 6.45, *p* = 0.015, and CXCL12, *F*(1,38) = 4.23, *p* = 0.047, as assessed by a linear model using the presence of ICAM1, SDC4, and CXCL12 as independent variables, and donor ID as a covariate of no interest to account for global donor effects. Each data point represents the mean number of adherent cells per device. One device was used per condition per donor. Under the hypothesis that the SDC4 effect would be dependent on its ability to immobilize CXCL12, we created an additional model to test for an interaction between CXCL12 and SDC4 model terms, but surprisingly this was not the case *F*(1,37) = 0.86, *p* = 0.36.

We then sought to further characterize the effects of the coating molecules on adhesion to the sidewall versus the center of the channel. To achieve this, we dilated the image of the channel edge slightly to make a sub-ROI encompassing wall adherent leukocytes. Leukocytes in the remainder were taken to be mid-channel, i.e. top and bottom wall adherent. This approach (Fig. 5c) more clearly demonstrated the trends seen in the heatmap: adhesion probability for wall associated leukocytes peaks sharply after the expansion and then diminishes as flow becomes established. This comes in contrast to channel center adhesion, which exhibits a small transient peak at the expansion, and then overall steadily increases as flow stabilizes. Wall associated adhesion then peaks again at the beginning of the expansion, as wall associated leukocytes are again forced to collide with overriding erythrocytes. Adhesion within constrictions and expansions themselves was minimal.

Looking at the channel cross section served to validate the sub-ROI approach (Fig. 5d): wall-associated cell adhesion probability peaked sharply at the sidewalls, with a tail attributable to the sidewalls of the expansion and constriction, while channel center adhesion was zero at the walls.

With these definitions in hand, we then performed spatial parametric modeling to test whether the effects of adhesion molecules were dependent on transitional vs. established flow. As initial validation, we looked at these effects across the center of the channel for leukocytes that were not wall adherent (Fig. 5e). As expected, leukocyte adhesion under these conditions was strongly ICAM-1 dependent, with a peak at the center of the channel where shear stress is maximal, and the leukocyte is more dependent on firm adhesive contacts to avoid detachment. The effects for ICAM-1 dominated over those of CXCL12 and SDC4 in this fine-grained analysis but CXCL12 did reach a small but significant positive peak in the center of the channel.

Having obtained these results from the cross section of the channel, we then analyzed patterns down the entire length of the ROI in response to expansion, flow establishment, and constriction.

Given that the expansion is an additional factor that influences adhesion but is not captured by our statistical model, we expected that in modeling, the effects of coating molecules would be lowest immediately after the expansion, i.e. that the expansion effect would be captured in a larger error (unexplained variance) term within this region. To our great surprise, this was not the case. As shown in Fig. 5f, the effect of ICAM-1 coating was greatest immediately after the expansion. Therefore, while the effect is driven by erythrocytes, it is dependent on ICAM-1. For these wall-associated leukocytes, the effects of CXCL12 and SDC4 failed to reach statistical significance at any point.

To further contextualize this unexpected result, we fit the same model for leukocytes in the center of the channel (Fig. 5g). In contradistinction to wall adherent leukocytes, all three coatings contributed to channel center adhesion immediately after the expansion, with the ICAM1 effect being relatively minimal. This changed over the length of the ROI as flow stabilized and the dependence on ICAM1 steadily increased.

### Expansion-induced leukocyte adhesion is dependent on LFA-1

**Figure 6:**
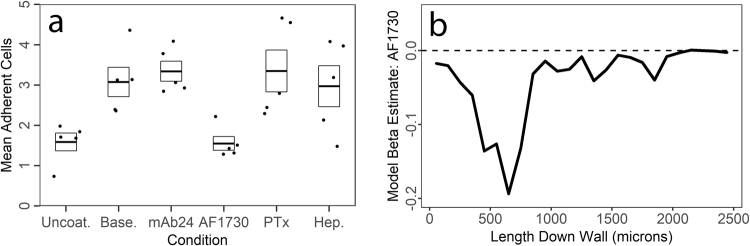
Leukocyte adhesion as a function of treatments and varying extensional stresses. (**a**) Mean number of adherent cells per ROI in response to LFA-1 activating antibody mAb24, LFA-1 blockade antibody AF1730, pertussis toxin, and Heparinase III. Of these, only AF1730 had a significant effect. Each point is the mean value for one participant. N = 5 donors. Boxes indicate mean and standard error. Isotype controls and untreated channels are averaged into ‘baseline’ for display clarity purposes – statistics were calculated for antibodies vs. paired isotype. (**b**) Spatial linear model fit for the contribution of AF1730 to adhesion probability for wall adherent leukocytes in straight channels. The strongest effect of AF1730 blockade of LFA-1 is observed immediately after the expansion.

To further test this phenomenon, and to establish the sensitivity of our microfluidic models, we tested the effects of various functional treatments on adhesion (Fig. 6a). For this, we chose to manipulate the integrin LFA-1, for which ICAM-1 is the ligand, by both the activating antibody mAb24 that stabilizes the active state of the integrin, and AF1730, which blocks its binding to ICAM-1. In addition to this, we investigated the effects of one chemokine receptor signaling pathway by blocking G alpha i activation with pertussis toxin, and the effect of glycosaminoglycans on SDC4 by cleaving them with heparinase III. These effects were interrogated using paired t tests for each treatment versus its isotype control (in the case of anti LFA-1 antibodies) or untreated, baseline adhesion (in the case of pertussis toxin and heparinase III). Since there were no differences between untreated and isotype control adhesion, these control conditions are summarized as ‘baseline’ for simplicity in Fig. 6a. AF1730 treatment reduced adhesion significantly, *t*(4) = 4.24, *p* = 0.013, while mAb24 had no effect, *t*(4) = 0.65, *p* = 0.55, consistent with maximal or near-maximal LFA-1 activation in this model. Pertussis toxin did not show any effect on adhesion, *t*(4) = 0.41, *p* = 0.7, which is consistent with other published studies showing that leukocyte arrest is not G alpha i dependent, especially *in vivo*^51,52^. Finally, heparinase III did not decrease adhesion, *t*(4) = 0.34, *p* = 0.7, but it also failed to reverse the SDC4-dependent increase in CXCL12 immobilization under our treatment protocol.

In this set of experiments we also assessed whether LFA-1 blockade by AF1730 would mirror the observed ICAM-1 dependence of wall associated adhesion in response to the expansion by fitting a model to AF1730 vs. isotype data from straight channels in the devices. This model used AF1730 treatment in our covariate matrix, along with donor as a covariate of no interest. To our amazement, the effect of AF1730 was almost entirely restricted to the initial expansion and the area immediately after the expansion (Fig. 6b), indicating that LFA-1-ICAM1 interactions mediate the effect of post-capillary venule expansion mimicry in our model.

### Effects of erythrocytes on increasing adhesion efficiency are not attributable to viscosity

By resuspending leukocytes in the presence of a physiological concentration of erythrocytes, we also increase the viscosity of the cell suspension. To prevent any confounding effect of varying viscosity on shear stress, we carefully chose a gravity-driven, pressure-controlled model rather than a syringe-pump driven, volume-controlled model. However, simple kinetic effects of speed could also potentially allow leukocytes more time to establish firm adhesive interactions. To address this concern, we investigated leukocyte adhesion in media with and without erythrocytes, and added to the clear media the polymer polyvinylpyrrolidone of 40 kDa molecular weight (PVP-40). PVP-40 is an established method of increasing media viscosity, and we were able to recreate typical viscosity of leukocyte-erythrocyte suspensions using a final concentration of 10% weight/weight PVP-40. For this form of PVP-40, that constitutes a 2.5 micromolar concentration that is osmotically insignificant.

**Figure 7:**
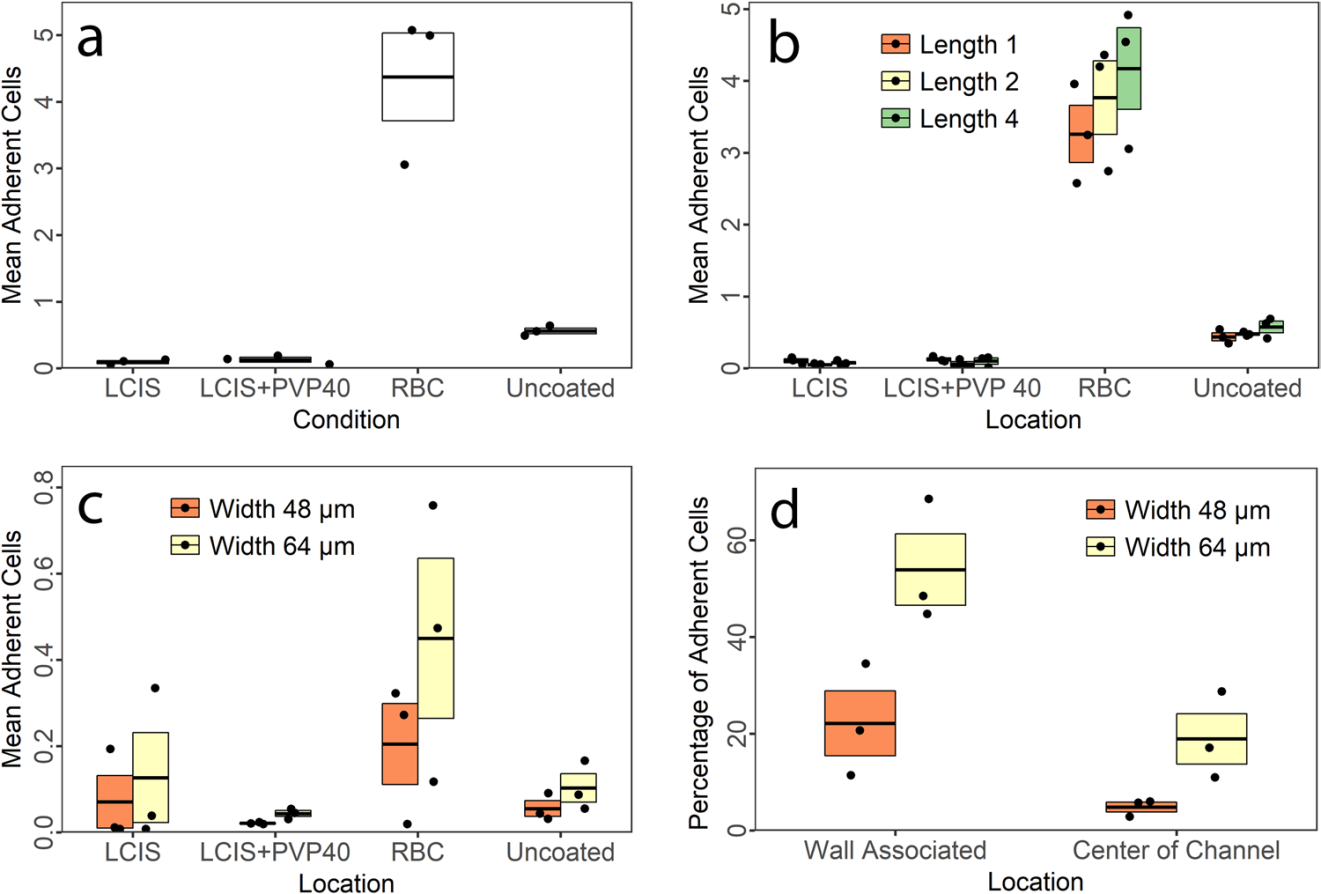
Leukocyte adhesion as a function of media viscosity in channels of physiological scale. (**a**) Mean number of adherent cells per ROI for leukocytes resuspended in media (LCIS), media with viscosity experimentally increased using polyvinylpyrrolidone (PVP-40), and media reconstituted with erythrocytes (RBC). Significant adhesion only occurs in the presence of erythrocytes. Data are from the devices displayed in Supplemental Fig. 1B. Each point is the mean value for one participant. N = 3 donors. Boxes indicate mean and standard error. (**b**) The same data as in A, demonstrating ROI length-adhesion relationship. Length 1 ROIs with the highest extensional stress did not induce a higher adhesion probability than length 2 or length 4 with the lowest adhesion probability. (**c**) Mean number of adherent cells per ROI in the same conditions as in A, with the same donors as in A, but using a device of 16 micrometer channels, expanding to 48 vs. 64 micrometer expansions (Supplemental Fig. 1C). (**d**) Leukocyte adhesion probabilities as a function of wall collision and shear stress in devices at physiological scale. Shear stress is highest in the center of the 48 micrometer expansions and lowest at the walls of the 64 micrometer expansions. However, critically, shear stress is higher on the wall of the 48 micrometer expansion than in the center of the channel in the 64 micrometer expansion, demonstrating that induced wall collisions can moderate the effects of higher shear stress in channels of physiological scale.

Increasing media viscosity with PVP-40 did not rescue the leukocyte adhesion deficit in clear media, nor did it have any statistically significant impact on adhesion, *F*(1,6) = 0.74, *p* = 0.42, (Fig. 7a).

Version 2 of the device (Supplemental Fig. 1B) was used to assess whether higher extensional rates induced by shorter expansions would further increase adhesion. As evident in Fig. 7b, this was not the case – length 1 ROIs with the strongest expansion forces had lower adhesion than length 4 ROIs. We believe this to be due to the longer length and therefore greater territory for adhesion in length 4 ROIs. Simple line fitting to account for the extra length is unfortunately not informative, since the variable length occurs in the higher shear expansion and constriction regions, and is therefore impossible to normalize. Of further interest to us in establishing a reliable model for quantification, we also tested signal-noise ratios in the different types, using the observable effects of coating vs. no coating and RBC vs. LCIS. These did not differ repeatably between types, indicating that all ROIs are equally capable for biological experimentation.

We tested these same effects with the same samples in a third device design, using channels at the larger end of true physiological scale and observed the same effect, although overall adhesion in these devices was lower still (Fig. 7c).

### In channels of physiological scale, expansion phenomena dominate over shear stress in determining relative adhesion probability

Thanks to progress in our device designs (Supplementary Fig. 1), we were able to create a leukocyte adhesion model that features post-capillary expansion mimicry on the upper end of physiological scale (48 and 64 micrometers). These expansions were specifically designed to test the relative effects of shear stress versus expansion. Within these ROIs, the shear stresses are (in descending order): 48 micrometer sidewall >64 micrometer center >48 micrometer center >64 micrometer sidewall. Therefore, if shear stress is the principal determinant of adhesion probability at this scale, adhesion would be predicted to be higher in the center of the 64 micrometer ROIs than on the sidewalls of the 48 micrometer ROIs. However, this was not the case (Fig. 7d). Despite higher shear stress on the walls of the 48 micrometer ROIs, adhesion here matches that of the center of the 64 micrometer ROIs, demonstrating that the effects of expansion and erythrocyte collisions can counteract higher baseline and adhesion-induced peak shear stresses in these channels at physiological scale.

## Conclusions

Here we have presented a device and analysis method for systematic investigation of leukocyte adhesion in response to rheological forces at the capillary to post-capillary venule transition.

Initial experiments with leukocytes in media alone without erythrocytes suggested that extensional stress itself could modulate leukocyte adhesion, independent of margination. However, this effect was later found to be dependent on leukocyte-leukocyte doublets or aggregates, as pre-filtered leukocytes did not show such an effect, and exhibited very low adhesion probability. Notably, we do not believe this is due to cell depletion, since manual counting demonstrated no detectable change in cell number with filtration, and inclusion of erythrocytes together with filtered cells recovered the deficit. We propose that this phenomenon may be due to a jamming effect as anisotropic particles, such as leukocyte doublets or aggregates, are reoriented under conditions of extensional flow^53^.

Inclusion of erythrocytes at physiological hematocrit induced marked margination and slow rolling of leukocytes on the channel sidewalls, and greatly increased leukocyte adhesion probability while significantly shifting adhesion from the center to the sides of the micro-channel. Additionally, the presence of erythrocytes significantly attenuated the effects of secondary capture of leukocytes.

Integrating across the three cell conditions - raw leukocytes, filtered leukocytes, and filtered leukocytes with erythrocytes - by far the strongest predictor of leukocyte adhesion was forcing against the wall by cell-cell interactions, either as a result of aggregate jamming (as in the case of unfiltered leukocytes) or by intravascular collisions between erythrocytes and leukocytes. The forces exerted on marginated leukocytes by erythrocytes are overwhelming, on the order of several hundred pico-newtons^54^. In comparison with these effects of cell-cell interactions, the effects of shear and extensional rates and stresses *per se* were less significant. This point was further underscored by the inability of increasing viscosity to rescue the adhesion deficit of leukocytes in erythrocyte-free media.

Though extensional flow induced cell-cell forcing at the sidewalls, we did not observe a strong response of adhesion to increasing extensional stress. In fact, adhesion was highest in length 4 ROIs with the lowest extensional stress, versus length 1 ROIs with the highest. This is potentially attributable to the larger physical size of length 4 ROIs, and therefore larger potential capture area. A linear fit of adhesion vs. ROI length does show length 1 ROIs to give higher-than-expected adhesion. However, because the variable length lies in the higher-shear and disturbed flow regions of the expansion and constriction, such an analysis is not informative. Future, more exhaustive studies of geometry versus adhesion could potentially define whether the effect exhibits a threshold phenomenon, a response to increasing extensional stress, or both.

Convergent data from spatial parametric modeling of the effects of coating, adhesion-stimulating molecules CXCL12, SDC4, and ICAM1 and the LFA-1 blocking antibody AF1730 led to the surprising observation that the erythrocyte-dependent effects of the expansions were critically dependent on interactions between LFA-1 and ICAM1. This could potentially be attributable to increased surface contact area from compression, inherent mechanosensitivity of integrins^55^ and/or to integrin-linked mechanosensitive pathways. These intriguing observations deserve further study.

Scaling biological assays down to microscale always introduces significant new statistical challenges, and this problem is compounded in the case of leukocyte adhesion with flowing media, where the rheological effects of adherent leukocytes become significant as channel scale approaches the same order of magnitude as leukocyte diameter. To overcome these problems, we greatly increased the number of observations and employed computer vision to allow us to make analyses that were not possible previously (e.g. 512 ROIs per device in the final design).

The inclusion of erythrocytes is necessary for adhesion assays at this scale. They increase adhesion probability to detectable level in the smallest channels and essentially eliminate secondary capture. We also observed fewer clogging events within the microchannels in the presence of erythrocytes. Altogether, the inclusion of erythrocytes in the leukocyte adhesion assay increased reliability, physiological validity, and statistical robustness.

There are several limitations and caveats of this work. First, our model uses the traditional photolithography, glass-and-PDMS Whitesides technique for device construction, generating essentially two-dimensional patterns. While this approach is expedient, it does not allow us to recapitulate the full three-dimensional rheological milieu at the capillary to post-capillary venule transition. However, we predict that in a fully three-dimensional system, where erythrocytes flow over newly entrant leukocytes, the effects of cell-cell contacts will be further increased, since streamlines from the joining capillary are extended across the vessel wall. Further technical advances in microfluidic device construction are necessary before such a junction can be reliably constructed at this microscale.

Second, our analysis workflow currently necessitates isolating leukocytes from whole blood in order to stain them with cell tracker dyes. Erythrocytes must then be re-introduced later in sample processing. While this has the potential to reduce experimental variance due to differences in plasma protein expression, and allow cell type specificity of effects (e.g. using isolated CD4+ T lymphocytes) it comes with risks. First, it introduces cells to significant stress, which can cause cell death, especially in neutrophils. Second, fluorescent staining of cells may increase their stiffness^56^. The specific staining protocol used should reduce this effect^57^, and margination and rolling were preserved for unstained cells in our system (Supplemental Movie 2). Nevertheless, an increase in stiffness would be predicted to promote margination^54,58^. Last, while it may reduce experimental variance between donors, depriving cells of plasma proteins may significantly affect their behavior.

Finally, our assay used immobilized adhesion molecules and chemokines rather than inflamed endothelial cells as an adhesion substrate. This was a deliberate decision, made in order to dissect potential leukocyte-intrinsic responses to rheological stresses. The inclusion of endothelial cells would have confounded results of effects of shear and extensional stress on leukocytes, versus as moderating factors in inflammatory activation of endothelial cells. With these data established, future experiments could include inflamed endothelial cells as the adhesion substrate.

Despite these caveats, we believe that the present study represents a significant first step in addressing the practical challenges inherent in scaling leukocyte adhesion assays down to physiological scale. We also believe that our results call for a reconceptualization of the effects of rheological stresses on leukocyte adhesion at *in vivo* scale. Whereas shear stress is the dominant factor determining leukocyte adhesion probability in larger channels, the effects of cell-cell interactions play a more important role in smaller channels.

## Methods

### Special Ethics Statement

As detailed below, the research involving human participants and animal subjects adhered to all institutional, local, state, federal, and international laws and guidelines concerning responsible and ethical conduct of research. Research involving mice was approved by the Case Western Reserve University Animal Resource Center (IACUC Approval Number: 2015-0118), which is accredited by the Association for Assessment and Accreditation of Laboratory Animal Care (AAALAC). Research activities strictly followed the procedures and protocols outlined in that approval. Research involving human samples was approved by the University Hospitals Cleveland Medical Center Institutional Review Board (UHCMC IRB Approval Number: 08-15-17), which is accredited by the Association for the Accreditation of Human Research Protection Programs (AAHRPP). Our research activities strictly followed the procedures and protocols therein. Most of the blood samples were obtained de-identified from the Case Western Reserve University Hematopoietic Biorepository & Cellular Therapy Shared Resource (HBCTSR), which also acts in accordance with its own UHCMC IRB approved protocols. Informed consent was obtained by the HBCTSR for those participants. Other samples were obtained under our IRB-approved protocol. Informed consent was obtained by the experimenter, recorded, and stored separately in a secured location.

### *In vivo* imaging

C57BL/6-Tg(UBC-GFP)30Scha/J (Stock #004353) mice were obtained from The Jackson Laboratory (Bar Harbor, ME, USA). Animals were housed, bred, and handled in the Case Western Reserve University Animal Resource Center (IACUC) in accordance with approved Institutional Animal Care and Use Committee experimental protocols (IACUC Approval Number: 2015-0118) and maintained on a Teklad 2018 S alfalfa free diet (Harlan Lab, USA) starting two weeks before imaging to reduce autofluorescence. Animals were given open cranial window preparations as previously described^59,60^. Animals were imaged within five days of cranial window implantation to preserve inflamed vessel architecture. Mice were anesthetized with nebulized isoflurane (3% induction, 2% maintenance) in 1:1 O2:air and placed in a stereotactic holder. Mice were then placed in a custom environmental chamber and maintained at a constant animal temperature of 37 °C via both heating pads and other environmental controls. Temperature, anesthetic depth, and breathing rate (approximately 40–80 breaths per minute) were monitored to ensure animal health and comfort. A Leica SP5 confocal microscope equipped with a 20× water immersion lens (Leica HCX-APO-L, N.A. 1.0) and a tunable 16 W Ti/Sapphire IR laser tuned to 860 nm (Chameleon Coherent, Inc.) was used for intravital 2PLSM imaging. XYT images with an XY dimension of 456 × 456 µm were obtained at 71 ms intervals. The data were analyzed using Imaris (BitPlane, Inc.) to generate images.

### Device design and modeling

2D masks for photolithography and 3D models of devices for computational fluid dynamics (CFD) simulations were created using OpenSCAD (www.openscad.org). 3D model geometries were imported into ICEM CFD (ANSYS Inc., Canonsburg, PA), blocked, and meshed. Meshes were exported for Fluent (ANSYS) and fluid flow was modeled using incompressible flow, with an apparent viscosity^61^ of 1.85 cP, as a compromise value between 1.83 for vessels of 8 micrometer diameter, and 1.92 for vessels of 25 micrometer diameter. We used a pressure-based solver (PISO scheme, second order pressure, QUICK momentum). A realizable K-Epsilon model was incorporated after solution convergence in order to load the wall distance parameter into memory for our Fluent user-defined function in C. This did not result in any significant changes to model outcome, as consistent with the laminar nature of microfluidic systems.

### Photolithography

3″ silicon test wafers (University Wafer, Catalog #447, Boston, MA) were rinsed extensively with acetone and isopropyl alcohol, then placed on a hotplate at 300 °C for a minimum of two hours to drive off water. Wafers were then coated with SU8 2025 photoresist (MicroChem Corp., Westborough, MA) and spun on a WS-400-6NPP spin coater (Laurell Technologies Corporation, North Wales, PA) using empirical protocols. After soft bake, photoresist was exposed on a Karl Suss MJB3 mask aligner (Suss MicroTec, Garching bei München, Deutschland) using transparency masks (printed by CAD/Art Services, Bandon, OR) mounted on a quartz slide (Chemglass Life Sciences, Vineland, NJ). Wafers were then post-exposure baked according to MicroChem protocol and developed using SU8 Developer (MicroChem). The thickness of resultant channel masks was confirmed at multiple locations using a NewView 7300 optical profilometer (Zygo Corp., Middlefield, CT), and re-verified after device assembly with fluorescence imaging. To facilitate later release of molded devices, wafers were treated with 1% v/v Trichloro(1 H,1 H,2 H,2H-perfluorooctyl)silane (Sigma-Aldrich Corp., St. Louis, MO) in hexanes for 5–30 minutes, dependent on ambient humidity, at room temperature and washed extensively with isopropyl alcohol before overnight hard bake at 80 °C in a hybridization oven.

### PDMS layer

Polydimethylsiloxane (PDMS) Sylgard 184 (Dow Corning, Auburn, MI) was mixed at a 9:1 ratio of base to curing agent, centrifuged to remove bubbles, inverted extensively to mix base and curing agent, then centrifuged again before pouring over molds. PDMS on molds was then degassed for 4 hours in a vacuum desiccator before placing in a room temperature hybridization oven. The oven was then ramped to 80 °C and the PDMS baked overnight. After baking, the oven was turned off and allowed to return to room temperature before the PDMS and mold were removed. Devices were manually cut out using a craft knife. The center inlet was punched with a 1500 micrometer PDMS Port Creator (Cor Solutions, Ithaca, NY). The outer edge was cut under a dissecting microscope using a scalpel. Devices were pre-cleaned using Magic greener tape (3M, Catalog # 812, St. Paul, MN), and sonicated in 70% ethanol in a Bransonic (Danbury, CT) CPX1800H sonicator at 70% power for 15 minutes. PDMS layers were then placed within a biosafety cabinet, removed from the pouch, allowed to dry, and stored with tape on the channel side until bonding.

### Glass

40 mm round cover glasses (Warner Instruments, Catalog # CS-40R15, Hamden, CT) were sonicated at 100% power for 60 minutes in water. Sonicated cover glasses were treated with 1 molar potassium hydroxide for 1 hour, rinsed extensively in tap water, then de-ionized water, and finally washed in 70% ethanol before being allowed to dry in a biosafety cabinet and transferred to a sealed pouch until bonding.

### Bonding

Bonding was achieved by treating PDMS and glass in a Plasma Etch (Carson City, NV) PE-100-RIE at 30 W × 30 seconds @ 20 sccm O2 and 200 mtorr. PDMS and glass halves of the devices were then placed together in light conformal contact. Devices were then placed in a sterilization pouch and autoclaved for 40 minutes at 121 °C. The autoclave was left closed overnight, such that the devices remained at 80 °C for an additional 6+ hours to ensure maximal biocompatibility^62^.

### Device coating

Recombinant human CXCL12 and recombinant human Fc-ICAM1 chimeric fusion protein were obtained from R&D Systems (Minneapolis, MN). SDC4, expressed in mammalian cells so as to have glycosaminoglycans, was obtained from Adipogen Corporation (San Diego, CA). These molecules adsorbed onto the device walls at concentrations of 20, 80, and 50 micrograms per milliliter, for CXCL12, ICAM1, and SDC4, respectively, in phosphate buffered saline (PBS) for a minimum of four hours in a 37 °C incubator. After this incubation, device channels were blocked with 5% BSA in saline for a minimum of 30 minutes.

### Inhibitor Treatment

Pertussis toxin (Sigma Aldrich) was used at a final concentration of 100 nanograms per milliliter. Blocking antibodies AF1730 (R&D Systems) and mAb24 (Biolegend) were used at 20 micrograms per milliliter, as were mouse IgG1 isotype (GeneTex) and goat IgG isotype (R&D Systems). Heparinase III (R&D Systems) was used at 40 micrograms per milliliter. All inhibitors were added just before the experiment start, except for Heparinase III which was added to the initial coating solution.

### Blood collection and handling

Peripheral blood from healthy human donors was obtained fresh in heparinized tubes. Most blood was obtained de-identified from the HBCTSR. Some blood samples were obtained directly, using identical procedures and reagents, after acquiring informed consent, under our University Hospitals Cleveland Medical Center Institutional Review Board (IRB) approved protocol sample and data handling. Blood was centrifuged at 200 g for 10 minutes at 20 °C to sediment cells. Buffy coats were carefully aspirated from the top of the packed layer, mixed 1:1 with phosphate buffered saline, and then layered over FicollPaque PLUS 1.077 (GE). Cells over density media were centrifuged at 400 g for 40 minutes at 20 °C without brakes. The interface was isolated, diluted with PBS, and centrifuged again at 200 g for 10 minutes at 20 °C. During this time, cells were counted. After this centrifugation, cells were resuspended in 500 microliters of RPMI 1640 with 10% fetal bovine serum (FBS) and stained with CFDA-SE according to optimized protocols^57^. After the washing steps in this protocol, cells were finally resuspended at 4 million per mL in live cell imaging solution (LCIS, Thermo Fisher Scientific) supplemented with 5% BSA and 50 mM D-glucose. In indicated cases, cells were passed through a 35 micrometer cell strainer. Then, cells were mixed 1:1 with either LCIS, LCIS with 20% w/w PVP-40 (Sigma Aldrich Corporation) for a final concentration of 10% w/w (2.5 mM), or packed red blood cells drawn from the bottom of the centrifuged collection tube. Such mixing occurred immediately before experiments began to prevent erythrocyte aggregation. Cells were maintained at 37 °C during this final preparation.

### *In Vitro* Microscopy

Images were acquired on a Leica DMI 6000B with a heated environmental chamber stabilized at 37 °C (Okolab USA, Burlingame, CA). The objective used was Leica 5× NA 0.15 HCX PL S APO. Tiles were collected using L5 filter cube with 20% overlap between tiles, as leukocytes were still flowing.

### Image analysis

All images were stitched using MIST^63^, which is freely available as part of FIJI. Stitched images were then subjected to a custom-made MATLAB (MathWorks, Natick, MA) pipeline: for fast registration, images were downsampled to 18% of original size, then bandpass filtered using an order 8, 20 micrometer cutoff lowpass and an order 1, 250 micrometer cutoff highpass Butterworth filter. Phase congruency was then calculated using the Kovesi algorithm^43^, for which the MATLAB function is freely available. Input image contrast ranges were separately optimized for LCIS vs. RBC. Phase congruency images were then multiplied by the sine of the phase angle, or negative sine of the phase angle, depending on context (LCIS vs. RBC) to isolate channel edges. Resultant cleaned phase congruency masks were provided an initial manual alignment using control points, a step incorporated with data cleaning. Data cleaning took the form of marking and automatically excluding blocked lanes, or lanes with significant manufacturing debris. Devices with more than ten percent excluded lanes were excluded entirely. Further refined alignment of the data to the templates was accomplished using affine transform with Mattes mutual information. ROIs, with a 15 micrometer buffer zone, were extracted and rotated to common space, where cells were segmented automatically.

### Automatic cell segmentation

Raw images were transformed to template space, then highpass filtered using an order 4, 25 micrometer cutoff Butterworth filter to isolate cell information. Phase congruency and phase angle of the filtered image were calculated using Kovesi’s algorithm, using a minimum wavelength of 4 micrometers for phase congruency and 8 micrometers for phase angle. Seed regions were identified by thresholding the phase congruency image at phase congruency greater than zero. Potential regions for expansion were identified by median filtering the phase angle image using a 5 micrometer kernel, then automatically thresholding using Otsu’s method. Morphological reconstruction using these seeds and regions was performed using imreconstruct in MATLAB. This method robustly identified both flowing and adherent leukocytes, a small number of contaminating platelets, and rings of high pass filter artifact. This was further filtered to adherent leukocytes by enforcing an Euler number of 1 to remove rings, a major axis length between 5 and 25 micrometers to exclude flowing and rolling leukocytes, and an area above 30 square micrometers to exclude platelets. Centroids of the resulting segmented areas provided cell position and cell number on a cell-by-cell and ROI-by-ROI basis. Cell positions were visualized by kernel density estimation with an 8 micrometer Gaussian kernel.

### Statistics

Analysis of cell numbers was performed in R^64^ in R Studio^65^. Initial attempts at quantification used an ROI-by-ROI basis, with negative binomial hurdle models via the pscl package^66^ and diagnostic rootograms via the countreg package^67^ to handle the over-dispersed and zero-inflated count data. While this approach was viable, it was ultimately more robust to use mean number of adherent cells per ROI per device. These means were log-transformed to normality to satisfy linear model assumptions. In Figs 4–7, data were fitted using linear models as functions of experimental conditions and donor identification number as a covariate of no interest to account for donor effects. These models were analyzed with analysis of variance (ANOVA). In Fig. 6, paired samples t-tests for antibodies mAb24 and AF1730 were performed separately versus their isotype controls mouse IgG1 and goat IgG, respectively. Results of statistical tests are reported in the text with the type of test (e.g. *F* or *t*), followed by degrees of freedom in parentheses, the test statistic, and the *p* value. One device was used per condition.

For spatial parametric mapping in Figs 5 and 6, mean number of adherent cells were binned into 100 micrometer wide bins for analysis along straight ROI lengths, and 20 micrometer wide bins for analysis across straight ROI widths. These means were log transformed to normality, with an addition of one to account for zero data. These were then input into identical models as for overall means: linear models using donor identification number as a covariate of no interest. Beta terms, representing the least squares estimate of the contribution of the factors of interest to adhesion, were then reported. Additional 2D spatial parametric maps were generated in the same conditions, using 25 micrometer Gaussian-kernel smoothing of the data. These resulted in the same conclusions, but were not efficient to present visually.

Plots were generated using package ggplot2^68^. Reports were generated using knitr^69^.

### Data availability

The datasets generated and analyzed in this study are available from the corresponding author upon request.

## Electronic supplementary material

Supplemental Movie 1: Transiently Adherent Leukocytes on Inflamed Pial Vessels In Vivo

Supplemental Movie 2: Rolling Leukocytes in Unstained Whole Blood Flowing through Biomimetic Channels

Supplemental Materials
