# Supplementary material for: Biomimetic post-capillary venule expansions for leukocyte adhesion studies
Source: Sci Rep. 2018 Jun 19;8:9328. doi: 10.1038/s41598-018-27566-z (PMC6008471; doi:10.1038/s41598-018-27566-z)
Supplement: Supplementary file 3 — Supplemental Materials [file 41598_2018_27566_MOESM3_ESM.pdf]

Supplemental Information For:

Biomimetic post-capillary venule expansions for leukocyte adhesion studies

Bryan L. Benson, Lucy Li, Jay T. Myers, R. Dixon Dorand, Umut A. Gurkan, Alex Y. Huang and Richard M. Ransohoff

Supplemental Equations 1: Rotation of Basis

ANSYS Fluent provides velocity and velocity gradients in Cartesian basis (x,y,z):

$$E_3 = \{e_1 , e_2, e_3 \} \; (1)$$

To describe flow dynamics as relevant to rolling leukocytes, we must describe these velocity gradients relative to a new basis that is wall-relative:

$$M_3 = \{m_1 , m_2, m_3 \} \; (2)$$

We start with the wall distance at each cell. The gradient of wall distance provides one basis vector:

$$c = \nabla d \; (3)$$
$$m_3 = \hat{c} \; (4)$$

Then get the cross product of v and c, a vector tangential to the wall, orthogonal to both velocity and wall distance:

$$b = v \times c \; (5)$$
$$m_2 = \hat{b} \; (6)$$

The wall-relative rolling direction is the cross product of the wall gradient and wall tangent vectors:

$$m_1 = m_2 \times m_3$$

M_3 defines the wall-relative basis that can be used to compute a transformation matrix Q:

$$Q = \begin{bmatrix} m_1 \cdot e_1, & m_1 \cdot e_2, & m_1 \cdot e_3 \\ m_2 \cdot e_1, & m_2 \cdot e_2, & m_2 \cdot e_3 \\ m_3 \cdot e_1, & m_3 \cdot e_2, & m_3 \cdot e_3 \end{bmatrix}$$

The second order velocity gradient tensor S is then transformed to the new basis via:

$$[S'] = [Q][S][Q^T]$$

Extensional strain rates are then quantified as S_{ii} for extensional rates in m_i, where i=1:3

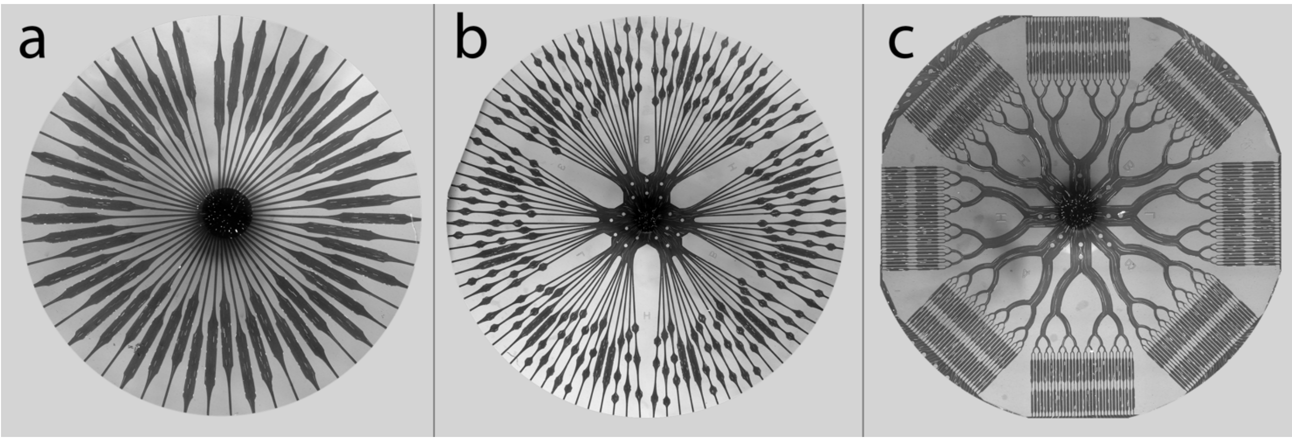

Supplemental Figure 1: Layout of devices used in the study

A) Device used in Figure 5: 54 straight ROIs around a circular manifold. Narrow inlets are 50 micrometers and expansions are 250 micrometers. Length 4 expansions are used on all.

B) Device used in Figure 4, 6, 7A and 7B: 240 ROIs in counterbalanced order around a circular manifold. ROIs are Length 1, 2, and 4 expansions with short straight sections, and a length 4 expansion with long straight section. Narrow and expanded channels have the same dimensions as in A. Branching using the planar adaptation of Murray's law reduces nonspecific adhesion at branch points

C) Device used in Figure 7C and 7D: 512 ROIs constituting a 16 micrometer narrow section and either 48 or 64 micrometer expanded section, in 256 rows of 2 in counterbalanced order. As in B, a branching pattern is critical in reducing nonspecific adhesion.

Supplemental Movie 1 Caption: Transiently Adherent Leukocytes on Inflamed Pial Vessels *In Vivo*

Cranial window imaging of Ubiquitin C GFP reporter mice in inflamed pial vasculature, demonstrating foci of leukocyte adhesion after sudden vessel expansions. 1X speed.

Supplemental Movie 2 Caption: Rolling Leukocytes in Unstained Whole Blood Flowing through Biomimetic Channels

Brightfield imaging of unstained leukocytes in whole blood, rolling on ICAM-1 coated surfaces of the biomimetic device. This demonstrates that slow rolling is dependent on ICAM-1 and erythrocyte contact, but not dependent on leukocyte staining. 1X speed.
